# High-resolution spectroscopy of [H,C,N]^+^: I. Rotationally resolved vibrational bands of HCN^+^ and HNC^+^

**DOI:** 10.1039/d5cp04201a

**Published:** 2025-12-29

**Authors:** Philipp C. Schmid, Samuel J. P. Marlton, Weslley G. D. P. Silva, Thomas Salomon, János Sarka, Sven Thorwirth, Oskar Asvany, Stephan Schlemmer

**Affiliations:** a I. Physikalisches Institut, Universität zu Köln Zülpicher Str. 77 50937 Köln Germany schmid@ph1.uni-koeln.de schlemmer@ph1.uni-koeln.de

## Abstract

Rovibrational spectra of the open-shell linear cations HCN^+^ (X̃^2^Π) and HNC^+^ (X̃^2^Σ^+^) are measured with leak-out spectroscopy in cryogenic 22-pole ion traps. The fundamental *ν*_1_ C–H stretching vibration of HCN^+^ is found at 3056.3412(1) cm^−1^ and the lower energy Renner–Teller (RT) component (Σ) of the *ν*_1_ + *ν*_2_ combination band is found at 3340.8480(2) cm^−1^. The resulting effective RT vibrational frequency of ≈300 cm^−1^ inferred from the comparison of these two bands indicates a large Renner–Teller splitting for HCN^+^. For HNC^+^, the *ν*_1_ N–H stretching vibration is found at 3407.9136(4) cm^−1^, much higher than expected from previous matrix work. Thanks to the rotational resolution of these infrared measurements, spectroscopic constants for the electronic fine-structure, molecular rotation, centrifugal distortion, *Λ*-doubling and spin-rotation interaction have been determined for the vibrational ground and excited states with high confidence. The infrared spectrum of HCN^+^ is rather rich and contains more bands including, *e.g.*, the electronic Ã ← X̃ transition. The analysis of this band and the pure rotational spectrum of HCN^+^ will be the subject of further publications.

## Abstract 2

Ro-vibrational spectra of the open-shell linear cations HCN^+^ (X̃^2^Π) and HNC^+^ (X̃^2^Σ^+^) are measured with leak-out spectroscopy in cryogenic 22-pole ion traps.

## Graphical abstract

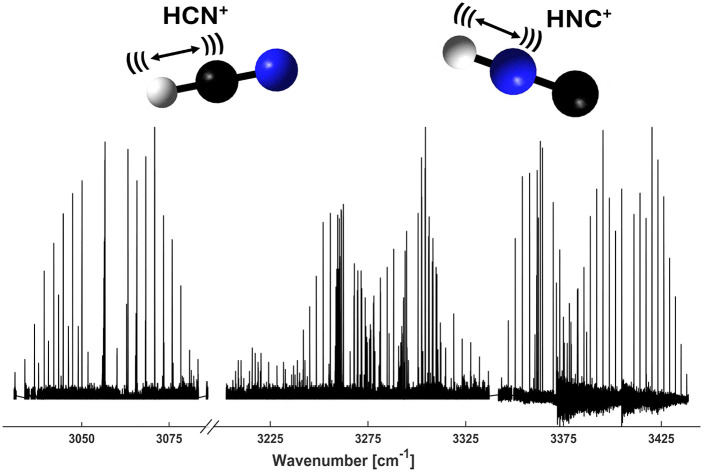

## 1 Introduction

The cyano group, –C≡N, is one of the key functional groups in organic chemistry. In space an impressive number of nitriles (R-CN) have been detected using radio astronomical techniques, ranging from simple linear cyanoacetylenes,^1,2^ to complex branched alkyl species such as isopropyl cyanide^3^ and even very heavy cyano-functionalized polycyclic aromatic hydrocarbons.^4,5^ In particular, prototypical hydrogen cyanide, HCN, and its metastable isomer HNC, serve as important diagnostic tools in astrochemistry. Consequently, their molecular properties, chemical reactivity, and spectra have been studied extensively, both theoretically and experimentally.^6^

In contrast, spectroscopic data on the corresponding radical cations, R-CN^+^ and R-NC^+^, are sparse. Specifically, very little is known from high resolution spectroscopy. Although HC_3_N^+^, HC_5_N^+^ and HC_7_N^+^ have been identified astronomically very recently from their pure rotational spectra,^7,8^ no high resolution studies of the parent species H-CN^+^ and H-NC^+^ have been reported to date. Previously, gas-phase studies on the reaction kinetics of HCN^+^ and HNC^+^ were conducted.^9–11^ In addition, important fundamental spectroscopic studies, particularly photoelectron spectroscopy (PES) measurements, were performed.^12–16^ Employing delayed pulsed field ionization photoelectron spectroscopy (PFI-ZEKE),^17^ the origin band and many vibrational bands of HCN^+^ were observed revealing a partially resolved rotational structure.

Understanding the spectra of these fundamental ions is also a challenge to quantum chemistry,^18–21^ as both isomers are linear open shell cations. Compared to the neutral case where HCN is the more stable isomer, the removal of one electron changes the energy ordering, making HNC^+^ the energetically most favorable species. HNC^+^ has a X̃^2^Σ^+^ ground electronic state whereas HCN^+^ has a X̃^2^Π electronic ground state.^22,23^ The latter fact was already revealed by PES-studies which determined the *Ω* = 3/2 F_1_-component state to be about 50 cm^−1^ lower in energy than the *Ω* = 1/2 F_2_-component. HCN^+^ is also subject to the Renner–Teller (RT) effect when excitation of the degenerate *ν*_2_ bending vibration is involved. Moreover, HCN^+^ has a low-lying Ã^2^Σ^+^ electronic state, first calculated by Köppel *et al.*^24^ to be comparable in energy with the fundamental *ν*_1_ C–H stretching frequency. The interaction of the X̃- and Ã-states along with the spin–orbit and RT couplings complicate the theoretical treatment of this fundamental ion. Nevertheless, especially for HCN^+^, vibrational energies as well as rotational constants were calculated by Peterson *et al.*^19^ and Tarroni *et al.*^21^ In order to challenge these calculations, experimental spectra at higher resolution than the PFI-ZEKE studies (≈1 cm^−1^) are required.

Conventional vibrational and rotational spectroscopy of reactive ions like HCN^+^ and HNC^+^ is hampered by the difficulty of supplying enough optical depth to record a spectrum. This difficulty has been overcome by a study in a Ne matrix,^25^ which reports frequencies for vibrational bands of the two target ions. However, no information on the molecular rotation is contained in these spectra.

Action spectroscopy in cryogenic ion traps allows overcoming the above limitations and studying the spectra of HCN^+^ and HNC^+^ in orders of magnitude higher resolution. In particular, the technique of leak-out-spectroscopy^26^ (LOS) has transformed ion spectroscopy into an almost straight-forward task where broad spectral coverage in high-resolution is paired with improved scanning speeds (see ref. 26–29 for examples) as will also be demonstrated in the current study.

This study is the first in a series of papers presenting ro-vibrational spectra of HCN^+^ and HNC^+^ (this study, paper I), followed by a report on pure rotational spectroscopy of HCN^+^ (paper II)^30^ using double-resonance spectroscopy based on the present work aiming to find HCN^+^ in space. In a third study (paper III)^31^ we report on the high resolution Ã ← X̃ electronic spectrum of HCN^+^. Each study focuses on different aspects of this fundamental molecular ion and its isomer.

In this paper we first describe the experimental method used to record high-resolution rovibrational spectra of both HCN^+^ and HNC^+^. In the Results section, we present an overview spectrum displaying several vibrational bands of HCN^+^ and one band of HNC^+^. Spectroscopic assignments will be performed based on the rotational structure of the different vibrational bands. The spectroscopic constants determined from fitting the effective Hamiltonians to the respective bands will be compared to the corresponding theoretically derived values. In the Discussion section, we will evaluate the results, connect the current work to the more detailed studies presented in work II and III and conclude with open questions for future spectroscopic work on HCN^+^ and HNC^+^.

## 2 Methods

The spectroscopic measurements of both, HCN^+^ and HNC^+^, were performed in the two Cologne cryogenic ion trap instruments LIRtrap^32^ and COLtrap,^33^ which have been described in detail before. In both apparatus primary ions were produced by electron impact ionization of methyl cyanide (Sigma-Aldrich) at an electron energy of about 100 eV. These ions are trapped inside a storage ion source^34^ and thus undergo further reactions with the neutral precursor gas prior to their extraction. The actual trap experiments are performed in cycles of typically 0.5 s (LIRtrap) or 1 s (COLtrap) period. At the beginning of each measurement cycle, a packet of ions is extracted from the ion source, mass selected for *m*/*z* = 27 in a quadrupole mass filter and injected into the cryogenic 22-pole ion trap.^35^

Upon trapping of the mass-selected ion ensemble, a short, high density (10^15^ cm^−3^) pulse of He is used to cool the translational motion as well as the internal degrees of freedom of the parent ions to the ion trap temperature *T*. These conditions were chosen differently, for HNC^+^: *T* = 40 K and for HCN^+^: *T* = 35 K in LIRtrap as well as *T* = 4 K in COLtrap, targeting different experimental goals as will be explained below. The rotationally resolved infrared spectra of HCN^+^ and HNC^+^ were recorded with LOS^26^ which uses a target gas to kick out the laser-excited parent ions from the trap. In detail, vibrationally excited cations can undergo a vibration-to-translation (V–T transfer) energy transfer process in collisions with the target gas (Ne or N_2_), thus gaining sufficient kinetic energy to overcome the longitudinal trapping barrier set by the difference between the trap potential and the exit electrode potential. For the HCN^+^ experiments, Ne was introduced in a continuous flow into the ion trap (LIRtrap). In COLtrap a 3 : 1 He : Ne mixture was used in a pulsed fashion to minimize freezing of Ne at the colder trap temperature. For the HNC^+^ LOS measurements in LIRtrap, a continuous flow of N_2_ was applied, for which the vibration-to-translation transfer in the LOS process was more efficient. Both the Ne and N_2_ densities of the continuous flows were on the order of a few 10^11^ cm^−3^ to yield optimal conditions as described earlier.^28^

After an initial 10 ms trap time without laser radiation, the ions were vibrationally excited by a continuous wave (cw) IR beam for a total time of 290 ms (LIRtrap). Ions experiencing a V–T transfer and thus leaving the trap are mass analysed (*m*/*z* = 27) by a second quadrupole mass filter and transferred to a Daly-type ion detector where they are continuously counted during irradiation. As a result, a signal is only recorded when a mass selected ion is vibrationally excited, which leads to almost background free spectra. At the end of the trapping cycle all remaining ions are ejected from the trap, in order to empty it prior to a new trapping cycle with a different laser frequency.

For the IR excitation, a high-resolution, high-power cw optical parametric oscillator (TOPO, Toptica) operating in the 3 µm spectral region was used. The laser beam was introduced into the apparatus through a CaF_2_ window along the longitudinal ion trap axis. After passing through the ion trap, the laser light exited through another CaF_2_ window where it was blocked. The power in front of the vacuum chamber was measured regularly to be several 100 mW throughout the measurements. For a direct measurement of the IR frequency, less than 1.5 mW of the light exiting the OPO was coupled into a wavemeter (Bristol Instruments, model 621 A-IR) with a spectral absolute accuracy on the order of 0.2 ppm. The laser frequency information was read-out in synchronicity with the recording of the LOS ion counts for each trapping cycle. By stepping the laser frequency in sufficiently small steps in between the trapping cycles, rovibrational spectra were recorded.

To supplement the experimental spectroscopic measurements, quantum chemical calculations were carried out using the electronic structure code CFOUR^36,37^ to determine the band positions of the *ν*_1_ fundamental of HNC^+^ and HCN^+^, as well as rotational constants in the ground and the excited states. Second-order vibrational perturbation theory (VPT2) in combination with coupled-cluster theory including single and double excitations^38^ and a perturbative treatment of the triple excitations, CCSD(T),^39,40^ has been utilized to calculate anharmonic vibrational frequencies using ROHF reference functions in the frozen-core (FC) approximation with augmented correlation-consistent basis sets up to the aug-cc-pV5Z level.^41,42^ In the case of HCN^+^, the strong Renner–Teller interaction requires the consideration of multiple electronic states to appropriately describe the bending fundamental. It is possible, however, to describe the *ν*_1_ stretching fundamental while considering only a single electronic state, which has been carried out here for the sake of simplicity. Therefore, for HCN^+^ we are only able to report the calculated band position without providing accurate rotational constants.

## 3 Spectroscopic background

**Fig. 1:**
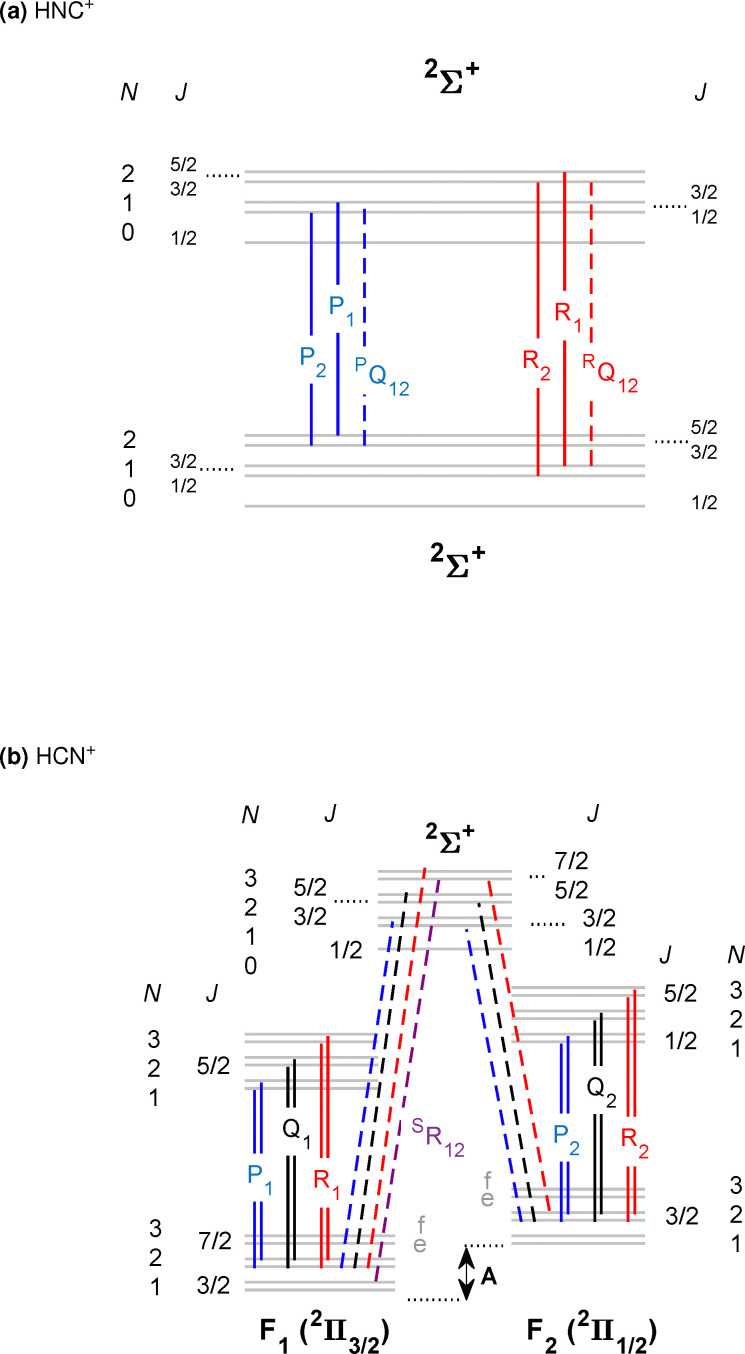
Schematic energy level diagrams for HNC^+^ and HCN^+^. Vertical lines depict observed rovibrational transitions belonging to typical vibrational bands of these two isomers. The upper part (a) illustrates a ^2^Σ ← ^2^Σ band and the lower part (b) a ^2^Π ← ^2^Π band (solid lines) as well as a ^2^Σ ← ^2^Π band (dashed lines). Also branch labels and angular momentum quantum numbers are indicated to aid the assignment of the experimental spectra.

For the spectroscopic assignment of the rotationally resolved vibrational spectra presented in this work, the rotational structure of the energy term diagrams of the ground and excited vibrational states of the two [H,C,N]^+^ isomers and the respective dipole transition selection rules are required. Fig. 1a and b display term diagrams for the X̃^2^Σ^+^ and the X̃^2^Π ground electronic states of HNC^+^ and HCN^+^, respectively. For HNC^+^ we observe the *ν*_1_(σ) fundamental N–H stretching vibration. Therefore, a ^2^Σ^+^ ← ^2^Σ^+^ transition as depicted in Fig. 1a is expected. For this *Λ* = 0 case, molecular rotation (angular momentum *R* [= N]) and electron spin (*S*) couple to the total angular momentum *J* following the rules of Hund's case (b). As such the energies of the rotational levels are given to first approximation by the terms^43^*F*_1_ = *B*_[v]_*N*(*N* + 1) + 1/2*γ*_[v]_*N**F*_2_ = *B*_[v]_*N*(*N* + 1) − 1/2*γ*_[*v*]_(*N* + 1)for *J* = *N* ± *S*, respectively. Here, *B*_[v]_ is the rotational constant of a vibrational state *v* and *γ*_[v]_ is the respective spin-rotation constant for that vibrational state. With the selection rules Δ*S* = 0, Δ*N* = ±1 and Δ*J* = 0, ±1 only a P and R branch will occur in the spectrum as indicated by the blue and red vertical lines in Fig. 1a. In fact, the Δ*N* = −1 transition (P-branch) consists of the three lines associated with the Δ*J* = 0 (Δ*S* = 0) and the two Δ*J* = −1 (Δ*S* = 0) components, of which the Δ*J* = 0 transition is low in intensity as indicated by a dashed line for this transition.

For HCN^+^ with *Λ* = 1 in the vibronic ground state, the angular momenta couple according to Hund's case (a), as depicted in Fig. 1b. The two fine-structure components *Ω* = 1/2 (F_2_) and 3/2 (F_1_) are well separated. For each component the rotational levels follow, to first approximation, the term^43^*F* = *B*_eff_*J*(*J* + 1)where *B*_eff_ ≈ *B* (1 ± *B*/*A*) with the larger value corresponding to the F_1_-component. Each rotational level is subject to *Λ*-type doubling with bigger (smaller) separations for the *Ω* = 1/2 (3/2) state.

Starting from this ground state energy structure, two typical vibrational bands are depicted in Fig. 1b. The lower energy transitions shown as the solid vertical lines depict a ^2^Π ← ^2^Π band while the dashed lines belong to a ^2^Σ ← ^2^Π band. The former case is expected for example for the *ν*_1_ fundamental C–H stretching band of HCN^+^ while the latter case holds for the *ν*_1_ + *ν*_2_ combination band of HCN^+^. Here, the excitation of a bending vibration (*ν*_2_-mode) is associated with a vibrational angular momentum *l* = 1 which gives rise to a set of vibrational states of which only one Σ component is shown in Fig. 1b for clarity. We display transitions of these two observed bands because already visual inspection shows that (i) the spin–orbit coupling constant *A*_0_ of the vibrational ground state as depicted in Fig. 1b can be determined from combination differences of specific rovibrational transitions (dashed lines) of the ^2^Σ ← ^2^Π band. In contrast, for the ^2^Π ← ^2^Π band the two fine structure components are not connected by rovibrational transitions. However, the energy difference of these transitions are (ii) a measure of the change of the fine structure constants Δ*A* = *A*_v_ − *A*_0_ for the vibrationally excited and the ground states, respectively. With the knowledge of these two quantities, *A*_0_ and Δ*A*, thus also *A*_v_ can be derived when two such bands are measured as in this work.

The above discussion shows that the spectra for the two isomers of [H,C,N]^+^ and also different vibrational transitions will display characteristic rotational band structures which will help in their unique assignment. Moreover, detailed spectroscopic constants for the corresponding ground and excited states shall be derived depending on the spectral resolution of the experiment. In the present study the PGOPHER program^44^ is used to determine these constants in a least-square fit procedure.

## 4 Results

### 4.1 Identification of vibrational bands

**Fig. 2:**
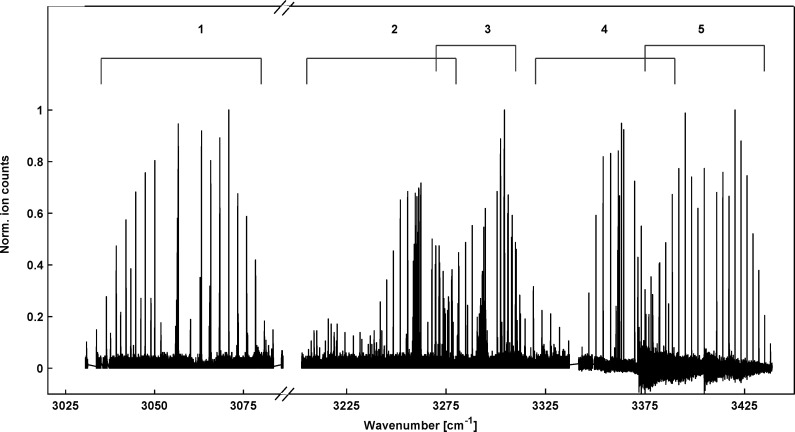
Overview of the infrared spectrum of molecular ions with mass *m*/*z* = 27. It is a collection of individual spectra recorded at different temperature conditions to illustrate the spectral coverage and the identification of the bands for HCN^+^ (1–4) and one for HNC^+^ (5) as marked by the numbered brackets and summarized in Table 1. Intensities of the individual spectra were normalized to allow for better representation.

The search for the vibrational bands of HCN^+^ and HNC^+^ was guided by the reported band positions in Ne-matrices,^25^ 3050 cm^−1^ and 3365 cm^−1^, respectively, and indeed bands were observed close to these positions. As it turned out, the spectrum in the 3 micron region is rather rich, containing many bands as shown in the overview spectrum in Fig. 2. Thanks to the cold temperatures in the ion trap, all bands are rather compact and can be numbered and assigned as summarized in Table 1 and Fig. 2. The band positions for HCN^+^ we find in this work agree very well with the theoretical predictions by Tarroni *et al.*,^21^ therefore, we use the same labelling of the bands in Table 1.

**Table 1:** Numbering and assignment of the various bands observed in the infrared spectrum shown in Fig. 2. Band positions ν are given in cm^−1^

Band	Species	Assignment^a^		Symmetry^a^	ν_exp_^b^	ν_theo_^c^	ν_theo_^d^	ν_theo_^e^
1	HCN^+^	X̃ (100)^0^		Π	3056	3058	3072	
2	HCN^+^	Ã (000)^0^		Σ^+^	3239		3233	
3	HCN^+^	X̃ (021)^1^	*κ*	Π	3273 f		3269	
4	HCN^+^	X̃ (110)^1^	*μ*	Σ^+^	3341^g^		3352	
5	HNC^+^	X̃ (100)^0^		Σ^+^	3408	3404	—	3404
6	HCN^+^	X̃ (110)^1^	*κ*	Σ^−^	—		3881	

a Assignment and symmetry of the upper vibrational state following the description of Tarroni *et al.*,^21^ Table III.

b This work as shown in Fig. 2. Further details on the bands are given below and in follow up work.

c This work, VPT2 at FC-ROHF-CCSD(T)/aug-cc-pV5Z.

d Band positions from Tarroni *et al.*,^21^ Table III.

e Band positions from Kraemer *et al.*,^45^ Table V.

f Only the upper *κ* band component lies in the observed range, for other *μ* component see Tarroni *et al.*,^21^ Table III.

g Only the lower *μ* band component lies in the observed range, for other *κ* component see Tarroni *et al.*,^21^ Table III.

The identification of the individual bands is primarily based on the rotational band structures as introduced in the previous section. Since several bands of the same type appear in the spectrum, the proximity of the corresponding band center to those predicted by Tarroni *et al.*^21^ has been used for the assignment. Apart from assigning features to vibrational bands of the two [H,C,N]^+^ isomers, also lines belonging to C_2_H_3_^+^ (another *m*/*z* = 27 ion emerging from the ion source) are found in the 3230 cm^−1^ range of the spectrum. But under the experimental conditions, only a few C_2_H_3_^+^ transitions were detected, with an intensity of less than 20% of the strongest peaks observed in the direct neighboring band 2 (Fig. 2). In the following, the fundamental C–H stretching band of HCN^+^ (band 1 in Table 1 and Fig. 2), a combination band for HCN^+^ (band 4) and the fundamental N–H stretching band of HNC^+^ (band 5) will be examined in greater detail to obtain spectroscopic parameters on both isomers. The very low-lying electronic Ã ← X̃ transition of HCN^+^ (band 2) will be further examined together with the other vibronic band (band 3) of HCN^+^ in a dedicated study (paper III).

### 4.2 The *ν*_1_ fundamental band of HCN^+^

**Fig. 3:**
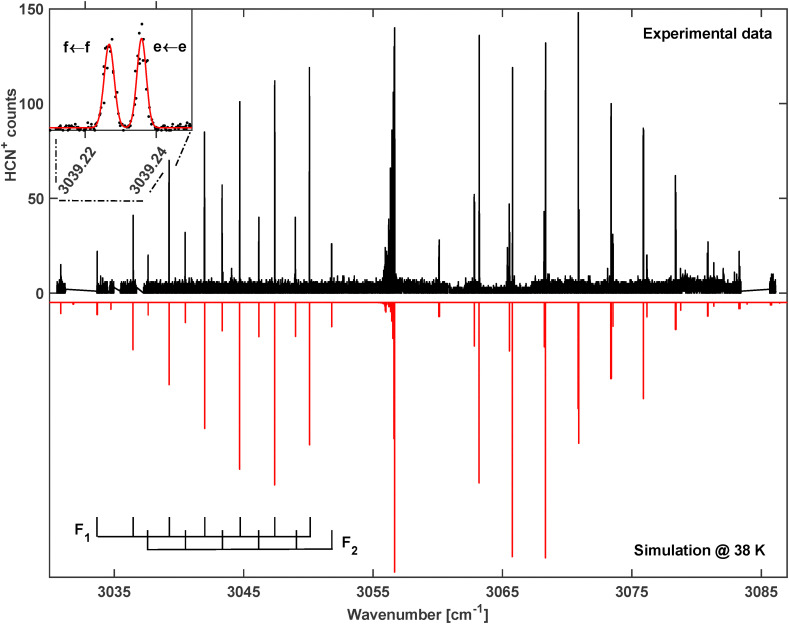
Rotationally resolved *ν*_1_ C–H stretching vibration of HCN^+^. Top: Experimental spectrum, measured at a trap temperature of *T* = 35 K. Bottom: Simulated spectrum at *T* = 38 K. Inset: *e*/*f* splitting of the *P*(6.5) transition.

Our detailed report starts with the *ν*_1_ fundamental band of HCN^+^ (band 1 in Fig. 2 and in Table 1) which is found close to the band assigned in Ne-matrices. Fig. 3 shows the recorded spectrum in greater detail at *T* = 35 K which consists of a clear P–Q–R structure with a band origin near 3056 cm^−1^. Inspection of the P-branch reveals two series of lines both with a clear 2*B* signature for a linear molecule. The two series show an alternating intensity pattern which varies with temperature. This is characteristic for two spin–orbit species as in the case of HCN^+^ with the two components ^2^Π_3/2_ (F_1_) and ^2^Π_1/2_ (F_2_). In Fig. 3 a wider gap between the P- and R-branches can be observed for the more intense lines. This is because the ^2^Π_3/2_ state with *J* ≥ 3/2 is lower in energy as already found by previous PES studies. This situation is depicted in the coarse term diagram of Fig. 1b. In fact, the band observed in Fig. 3 is typical for a ^2^Π ← ^2^Π transition which is expected for the *ν*_1_(σ) fundamental band. Lines belonging to the F_1_ (^2^Π_3/2_) and F_2_ (^2^Π_1/2_) species are observed as indicated by the F_1_/F_2_ rulers in Fig. 3. As a last sub-structure, each rotational line in the spectrum shown in Fig. 3 splits into two *Λ* components (see the inset in Fig. 3 for the resolved *Λ* doubling).

The rovibrational lines of the spectrum have been assigned and fitted using a standard Hamiltonian for a linear rotor with a ^2^Π electronic ground state as it is implemented in the PGOPHER program. The lower part of the graph in Fig. 3 shows the modeled spectrum at a temperature of 38 K where the observed line intensities agree rather well with the measured ones. The molecular parameters derived from this fit are shown in Table 2 along with current and previous calculations as well as earlier experimental results. A line list of the assigned transitions and fit residuals can be found in the SI.

**Table 2:** Spectroscopic parameters of HCN^+^ for the spectra shown in Fig. 3 and 4. Values are given for the ground state (^2^Π (0,0,0)), *ν*_1_ state (^2^Π (1,0,0)), and *ν*_1_ + *ν*_2_ state *μ* component (^2^Σ^+^ (1,1,0)), obtained by a global fit of the spectra. All values are in MHz, unless otherwise specified. Numbers in parenthesis represent the uncertainty of the last digits

Parameter^a^	This work (experiment)	This work (theory)	Peterson *et al.*^19^ (theory)	Tarroni *et al.*^21^ (theory)	Wiedmann *et al.*^17^ (experiment)	Forney *et al.*^25^ (experiment)
*B*_0_/cm^−1^	1.35275(1)		1.340(2)	1.3533	1.36(1)	
*D*_0_	0.100(3)	0.084				
*p*_0_	731.6(18)					
*q*_0_	−60.0(2)					
*A*_0_/cm^−1^	−49.3113(3)			−49.9	−49.8(2)	
*A*_*D*_0__	−43.1(3)					
*eQq*(^14^N)		−6.39	−6.50			
*μ*_A_/Debye		3.530	3.634			

*ν*_1_/cm^−1^	3056.3412(1)	3058.2	3099(35)	3071.9		3049.9
*B*_1_/cm^−1^	1.343387(9)			1.3445		
*D*_1_	0.088(2)					
*p*_1_	858.1(17)					
*q*_1_	−70.6(2)					
*A*_1_/cm^−1^	−48.5987(3)			−49.1		
*A*_*D*_1__	−40.9(2)					

*ν*_1_ + *ν*_2_/cm^−1^	3340.8480(2)			3352.0		3365.0^b^
*B*_1+2_/cm^−1^	1.36993(1)			1.3710		
*D*_1+2_	0.085(5)					
*γ*_1+2_	−137.1(8)					

rms/cm^−1^	4.0 × 10^−4^					

a For the two ^2^Π states, *p* and *q* are the *Λ* doubling parameters, *A* the spin orbit constants, and *A*_D_ its distortion constants. For the ^2^Σ state, *γ* represents the spin-rotation constant.

b In the original work this value was derived from a feature associated with the N–H stretch of HNC^+^.

In total, more than 80 transitions have been fitted in this spectrum which resulted in a very good agreement with an average obs-calc value of 4 × 10^−4^ cm^−1^. This good agreement indicates that the *ν*_1_ vibrational band is not perturbed and thus allows determination of its spectral parameters with high accuracy. As can be seen in Table 2, the calculated rotational constants of Tarroni *et al.*^21^ show excellent agreement with our data. They agree with the values from our fit to within 6 × 10^−4^ cm^−1^ for the vibrational ground state and 1.2 × 10^−3^ cm^−1^ for the *ν*_1_ vibrationally excited state which shows the accuracy of those high-level quantum chemical calculations. The band position, on the other hand, is 16 cm^−1^ off. Our calculated value at FC-ROHF-CCSD(T)/aug-cc-pV5Z level of theory agrees within 2 cm^−1^ with the experimentally determined value (see Table 2).

The experimental resolution is largely limited by the line width of the individual lines. A fit with a saturated Gaussian function^46^ on a well-isolated line, as *e.g.* the *Q*4.5(*e*) transition, results in an ion kinetic temperature of about *T* = 40 K. This temperature compares rather well with the nominal ion trap temperature of 35 K and the assigned rotational temperature of the fit.

### 4.3 The *ν*_1_ + *ν*_2_ combination band of HCN^+^

**Fig. 4:**
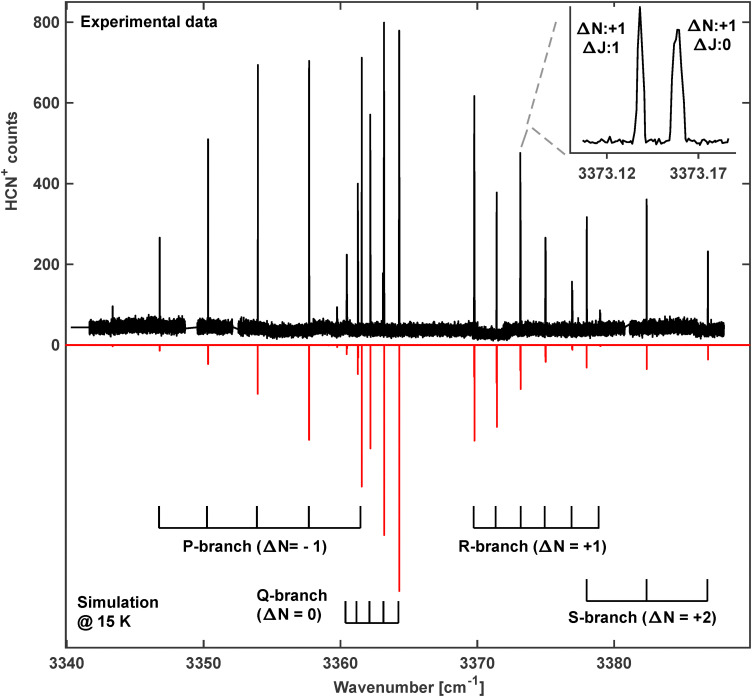
The rotationally resolved ^2^Σ^+^ ← ^2^Π combination band *ν*_1_ + *ν*_2_ of HCN^+^. Top: Experimental spectrum, measured at a trap temperature of *T* = 10 K. Due to the low temperature, the shown lines originate only from the lower-lying ^2^Π_3/2_ spin–orbit manifold F_1_ of the ground state. Inset: Spin-rotation splitting (*γ* constant) of the R1(3.5) transition. Bottom: Simulated spectrum at *T* = 15 K using parameters from Table 2. The brackets in the lower part indicate sub-bands as introduced in Fig. 1b.

Guided by the previous work in Neon matrices,^25^ another vibrational band was discovered around 3341 cm^−1^ (band 4 in Fig. 2 and Table 1), as shown in detail in Fig. 4. In Forney *et al.*^25^ this band was assigned to the fundamental *ν*_1_ mode of HNC^+^, a ^2^Σ^+^ ← ^2^Σ^+^ transition. But the close lying lines indicating Q-branch transitions do not support this idea but rather speak in favor of another carrier. Indeed, the band agrees very well with the theoretically predicted position of the *ν*_1_ + *ν*_2_ band for HCN^+^ at 3352.0 cm^−1^.^21^

The *ν*_1_(σ) + *ν*_2_(π) combination band for HCN^+^ carries vibrational angular momentum giving rise to two RT excited states Σ (^2^Σ^+^ and ^2^Σ^−^) as well as one RT state with *Δ* symmetry (split into ^2^*Δ*_5/2_ and ^2^*Δ*_3/2_). In the spectrum shown in Fig. 4 only the transition to the lower ^2^Σ^+^ component is observed. This band follows the transitions shown as dashed lines in Fig. 1b. In contrast, excitation of the N–H stretching vibration of HNC^+^ does not involve vibrational angular momentum, resulting in a ^2^Σ^+^ ← ^2^Σ^+^ band as discussed in Fig. 1a. This kind of band does not have a Q-branch and leads to a much simpler band structure than that observed in Fig. 4. The observed Q-branch in Fig. 4 indicates the involvement of several angular momenta such that also Δ*J* = 0 transitions are allowed. This leads to a clear assignment of this band to HCN^+^. Therefore, only the subtle rotational structure of the band, which is very different from the one of the *ν*_1_ fundamental N–H stretching vibration of HNC^+^, can tell us which band is observed.

In fact, in the experimental spectrum four different branches have been identified and are indicated by their change of the quantum number for the rotational angular momentum Δ*N* = −1, 0, 1 and 2 (see Fig. 4). The Δ*J* = Δ*N* = 0 branch is the already identified Q-branch and marked by a respective ruler in Fig. 4 as are the other branches too. Among those branches Δ*N* = 2 transitions are also possible for Δ*J* = +1 as also Δ*l* = +1 applies. Consequently, the line spacing in the Δ*N* = +2 branch is considerably larger due to the bigger steps in the rotational energies of the respective states as seen by the ruler for this branch in Fig. 4 (S-branch). In fact, this pattern is quite characteristic for such a ^2^Σ^+^ ← ^2^Π band.

The spectrum in Fig. 4 was recorded with the LIRtrap apparatus using similar settings as described above except for a lower nominal trap temperature of *T* = 10 K. As a consequence of the lower temperature all lines in this spectrum originate only from the lower-lying ^2^Π_3/2_ spin–orbit manifold of the ground state. In Fig. 4 the recorded rovibrational spectrum of the *ν*_1_ + *ν*_2_ combination band is shown together with a PGOPHER simulation based on the fitted parameters listed in Table 2. The simulated spectrum agrees very well with the observed spectrum which indicates the appropriate description of the band. The band position has been predicted by Tarroni *et al.*^21^ some 10 cm^−1^ higher in energy, still very close to the observation. Again, the calculated rotational constants show excellent agreement with our data.

The excited ^2^Σ^+^ state does not exhibit *Λ* doubling but due to the two orientations of the electron spin, the spin-rotation interaction (*γ*) splits many lines into close doublets. These are not discernible on the scale of Fig. 4 but are shown in an inset. The ^2^Σ^+^ ← ^2^Π transition also allows determining the spin–orbit constant *A* of the ground state since transitions from both ^2^Π_3/2_ and ^2^Π_1/2_ fine-structure states end up in the same ^2^Σ^+^ excited state. In order to obtain transitions from both components, the ion trap was heated up to about 40 K and several lines originating from the ^2^Π_1/2_ spin–orbit manifold were recorded. A complete line list can be found in the SI.

A global fit of the 48 transitions of the *ν*_1_ + *ν*_2_ band (32 originating from ^2^Π_3/2_, 16 originating from ^2^Π_1/2_) and those of the *ν*_1_ stretching vibration of HCN^+^ has been performed, the results of which are given in Table 2. This combined fit allows determining unambiguously several molecular parameters. The fine-structure constant of the ground-state, *A*_0_, is determined through the combination band which then fixes *A*_1_ when including the fundamental *ν*_1_ band. Likewise, the *Λ*-doubling constants of the ground state, *p*_0_ and *q*_0_, are set *via* the combination band and *p*_1_ and *q*_1_ again *via* the fundamental band. The average obs-calc deviation of the global fit is 4 × 10^−4^ cm^−1^. Thus, just these two bands helped to determine many spectral details of HCN^+^ with considerable accuracy. The parameters of the ground vibrational state have been refined further in observations of pure rotational transitions. Results of such measurements will be presented in one of the follow-up studies (paper II).^30^

Another band of HCN^+^ with a typical ^2^Σ^+^ ← ^2^Π structure has been found in our studies (see band 2 in Fig. 2), only about ∼100 cm^−1^ lower in energy as compared to the lower Σ component of the *ν*_1_ + *ν*_2_ combination band (band 4, Fig. 2). Because both bands exhibit a ^2^Σ^+^ ← ^2^Π spectrum, they are similar in their structure and rotational constants, and are therefore not easy to distinguish just based on this information. However, the lower RT component of the combination band has been predicted to be very close to the observed position of band 4. From the experimental data we find (*ν*_1_ + *ν*_2_) − ν_1_ = 285 cm^−1^, which is in good agreement with the calculated value of 294.1 cm^−1^ for the lower energy RT Σ component.^21^ Based on the good agreement of theory and experiment for our bands 1–3 and also for other bands observed by PES we first adopt this assignment of band 4. Observation of the higher lying Σ^−^ component and also the *Δ* components would solidify the assignment of bands 2 and 4 based on experimental data only. However, as will be discussed in the study on the electronic Ã ← X̃ transition (paper III),^31^ there is further experimental evidence for the current assignments of bands 2 and 4. This evidence is based on the highly accurate rotational constants, as well as the observation of hyperfine splittings observed for the electronic transition (band 2).

We take the agreement of the experimental and theoretical position of band 4 as an indication that also the separation of the two Σ components (bands 4 and 6 in Table 1) predicted by Tarroni *et al.*^21^ as 529 cm^−1^ to be quite accurate. This separation also agrees very well with that of the two Σ components of the X̃ (010)^1^ fundamental excitation of the *ν*_2_ bending vibration of HCN^+^ which lie at 294.1 and 821.5 cm^−1^, respectively. These values correspond to a bending frequency of *ω*_2_ = 573 cm^−1^ and a quite large RT parameter *ε* = 0.50 (see SI). Thus, in summary the experimental position of band 4 discussed in this section is strong evidence for a sizable Renner–Teller splitting of HCN^+^.

### 4.4 The *ν*_1_ fundamental band of HNC^+^

**Fig. 5:**
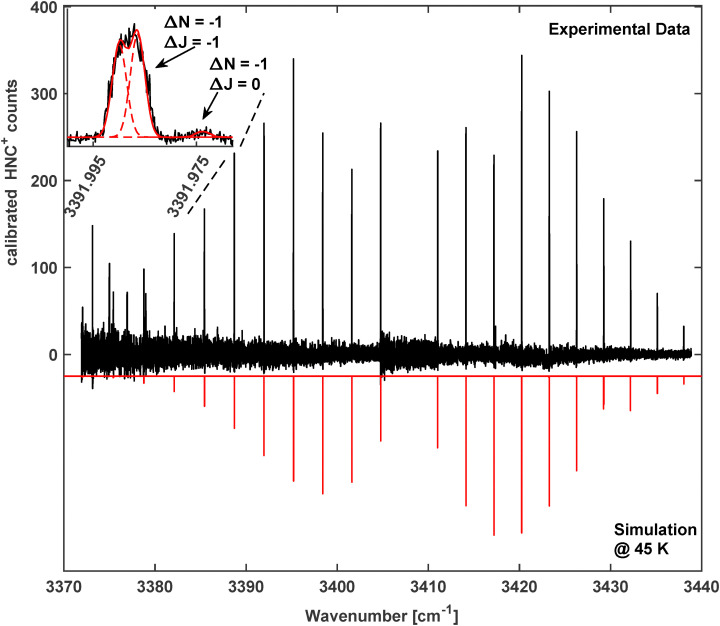
The rotationally resolved vibrational band of the fundamental *ν*_1_ N–H stretching vibration of HNC^+^. Top: Background corrected and intensity calibrated experimental spectrum at *T* = 40 K. Bottom: Simulated spectrum at *T* = 45 K using parameters from Table 3. Inset: P(6) transition with partially resolved spin-rotation splitting. On the left hand side of the spectrum the onset of band 4 in Fig. 2 can be seen.

In our search for the *ν*_1_ fundamental stretching vibration of HNC^+^, another compact band of the mass selected *m*/*z* = 27 species was observed around 3408 cm^−1^ (band 5 in Fig. 2 and Table 1). The spectrum was recorded at an ion trap temperature of *T* = 40 K and is shown in Fig. 5 together with another PGOPHER simulation. Partial freeze-out of the N_2_ collision gas in the trap led to drifts of the baseline counts which have been corrected by the “smooth baseline function” in PGOPHER. The detected counts were calibrated by adjusting the intensities of common lines in overlapping scans. This also led to the increased noise visible in Fig. 2 and 5.

We identify this spectrum as the rotationally resolved band of the *ν*_1_ fundamental stretching of the energetically lower-lying HNC^+^ isomer with a ^2^Σ^+^ electronic ground state.^9^ The experimental spectrum shows the typical attributes of a ^2^Σ^+^ ← ^2^Σ^+^ band, with a P-branch (Δ*N* = −1) and R-branch (Δ*N* = 1), while a Q-branch (Δ*N* = 0) is clearly missing. As detailed above, each rotational quantum level, *N* ≠ 0, is split into two spin-rotation components and consequently each rovibrational transition will split into three close lying lines with Δ*J* = 0, ±1, of which the Δ*J* = 0 component is typically very weak. Apparently the spin-rotation splitting is small and is only partially resolved as shown in the inset of Fig. 5, depicting a zoom of the P(6) triplet. Here the Δ*N* = −1, Δ*J* = 0 component has been resolved, while the two Δ*N* = −1, Δ*J* = −1 components are partially blended. This triplet structure has been fit using three individual Gaussian functions with a fixed width of 0.0014 cm^−1^ which corresponds to a Doppler temperature of about 50 K. The two Δ*J* = −1 components have been modeled with high intensities and the Δ*J* = 0 component with much smaller intensity, as seen by the dashed curves in the inset of Fig. 5. This behavior agrees with the expectations for the ^2^Σ^+^ ← ^2^Σ^+^ band as discussed above. Also, the missing Q-branch is a clear indication for the assignment of this band to HNC^+^. The band center differs significantly from the value of 3365 cm^−1^ reported by previous matrix measurements.^25^ Apparently this previous published study confused the *ν*_1_ fundamental band of HNC^+^ with the *ν*_1_ + *ν*_2_ combination band of HCN^+^ discussed in detail above.

Similar to the spectra of HCN^+^, the recorded spectrum was fitted in PGOPHER with a standard linear rotor Hamiltonian with a ^2^Σ^+^ electronic ground state to extract the spectroscopic parameters of HNC^+^. A total of 51 lines have been fitted and the resulting spectroscopic parameters are shown in Table 3. The corresponding model stick spectrum is displayed in the bottom part of Fig. 5. Furthermore, in this case the spectrum is very well reproduced by the model with only a small number of significant parameters, the band center, the rotational constants plus quartic centrifugal constants as well as the spin-rotation coupling constants in the vibrational ground and excited states. Similarly to HCN^+^, the calculated ground state rotational constant of Peterson *et al.*^19^ is in excellent agreement with our experimental data, the band position, however, is nearly 60 cm^−1^ off. In comparison, the value calculated by Kraemer *et al.*^45^ is within 4 cm^−1^, which also matches perfectly with our calculated value (see Table 3). The clear 2*B* harmonic structure of the spectrum shows that the constants do not change strongly in the vibrationally excited state. In particular, the spin-rotation constant only changes by about 5–6% which is the reason for the small observed splitting. As for HCN^+^, a line list of the assigned transitions can be found in the SI.

**Table 3:** Spectroscopic parameters of HNC^+^ for the spectrum shown in Fig. 5. Values for the ground state (^2^Σ (0,0,0)) and *ν*_1_ state (^2^Σ (1,0,0)) are given. All values are in MHz, unless otherwise specified. Numbers in parenthesis represent the uncertainty of the last digits

Parameter	This work (experiment)	This work (theory)	Peterson *et al.*^19^ (theory)	Kraemer *et al.*^45^ (theory)
*B*_0_/cm^−1^	1.57169(4)	1.566	1.571(2)	1.549^a^
*D*_0_	0.093(8)	0.092		
*γ*	86.9(34)			
*eQq*(^14^N)		−0.30	−0.39	
*μ*_A_/Debye		0.723	0.66	0.7

*ν*_1_/cm^−1^	3407.9136(4)	3404.0	3464	3404.19
*B*_1_/cm^−1^	1.55987(4)	1.554		1.538^a^
*D*_1_	0.088(9)			
*γ*	81.9(33)			

rms/cm^−1^	1.1 × 10^−3^			

a Value extracted from Table VI from the study by Kraemer *et al.*^45^

## 5 Discussion and conclusion

Cations and in particular open-shell cations like HNC^+^ and HCN^+^ are very reactive species and therefore difficult to study experimentally by conventional methods. Action spectroscopy in cold ion traps is the method of choice for vibrational, rovibrational and rotational spectroscopy of such elusive species. Our approach offers mass-selection, isolation, cryogenic cooling, and basically unlimited interaction time with light from high-resolution radiation sources.^47–51^ In particular, the development of the universal leak-out-spectroscopy method (LOS^26^) opened up a new chapter in these developments, as the named advantages can now be applied to many desired cationic or even anionic species, as demonstrated by many recent experiments.^27–29,50–57^

This study reports the high-resolution infrared spectroscopy of the HNC^+^ (^2^Σ^+^) and HCN^+^ (^2^Π) cations which triggered a subsequent overtone spectroscopy measurement of these two ions.^58^ Only very limited information on spectroscopic parameters is available to date, as the comparison in the tables of this work shows. The vibrational bands observed could be assigned safely to characteristic features which distinguish the electronic states of both species in the ground and vibrationally excited states. In fact, the three observed bands discussed in this work in detail are prototypical for a ^2^Π ← ^2^Π transition in the case of the *ν*_1_ fundamental band of HCN^+^, for a ^2^Σ ← ^2^Π transition in the case of the *ν*_1_ + *ν*_2_ combination band of HCN^+^ and for a ^2^Σ^+^ ← ^2^Σ^+^ transition in the case of the *ν*_1_ fundamental band of HNC^+^. While the first case is a typical Hund's case (a), and the latter is a typical Hund's case (b), the combination band is a transition from case (a) to case (b). It is interesting to see that all these cases appear for the two [H,C,N]^+^ isomers. The high quality of the experimental data allowed fitting the spectra to standard model Hamiltonians, and obtaining the spectroscopic constants for the ground states and the vibrationally excited states with high accuracy. Identifying the lower energy Σ^+^ state of the two RT components for the combination band and comparison with previous theoretical works confirm a sizable RT-parameter (*ε* ≈ 0.5) for HCN^+^. Extending the spectral coverage will reveal the missing RT components and unfold more details on the couplings of motions for this simple triatomic molecule.

In a continuation of the present work, the extracted ground state rotational constants of HCN^+^ allowed us to search and record pure rotational transitions of this molecule using a rotational–vibrational double-resonance scheme as described earlier.^52^ Results from this work will be the topic of an accompanying article (paper II)^30^ in this series of works on the isomers of HCN^+^. Rotational spectra of such open-shell species are complicated by hyperfine interactions and even Zeemann splittings. This challenge could be tackled by the increased resolution of our millimeter- and sub-mm-wave studies such that the work on HCN^+^ will facilitate a future search of this fundamental ion in space.

The combination of the ease of tunability of today's infrared radiation sources, like the commercial OPO system in this work and quantum cascade lasers as well as the exceptional fidelity of the LOS technique led to a tremendous increase in the scanning speed. Thanks to the broad band coverage of the above light sources a spectral coverage of 10–15 cm^−1^ can be recorded within a day in favorable cases. This enabled the recording of the rotationally resolved electronic Ã ← X̃ transition of HCN^+^ as only briefly discussed here in the overview spectrum. The analysis of this infrared spectrum is subject of a third publication in this series (paper III).^31^ This spectrum exhibits further partially resolved splittings which, together with the details of the rotational hyperfine structure of the ground vibrational state are taken as experimental evidence for the detection of this low-lying electronic transition.

Despite all the advancements thanks to LOS there are also many open questions associated with details of the experimental technique. For example, it is not clear to date, why spectroscopic detection of HNC^+^ required N_2_ as a target gas and did not work with Ne. However, the two isomers of [H,C,N]^+^ are so similar in their properties that further comparisons of the two systems might help to unravel more details on the efficiency of the vibration to translation energy transfer which is the basis of LOS.

## Conflicts of interest

There are no conflicts to declare.

## Supplementary Material

CP-028-D5CP04201A-s001

## Data Availability

Data are available from the authors upon request. The supplementary information (SI) is available and describes the terms of the Renner–Teller splitting and also includes the line lists of the spectra presented in the article. See DOI: https://doi.org/10.1039/d5cp04201a.
