# Supplementary material for: High-resolution spectroscopy of [H,C,N]+: I. Rotationally resolved vibrational bands of HCN+ and HNC+
Source: Phys Chem Chem Phys. 2025 Dec 29;28(7):4394–404. doi: 10.1039/d5cp04201a (PMC12851410; doi:10.1039/d5cp04201a)
Supplement: CP-028-D5CP04201A-s001 [file CP-028-D5CP04201A-s001.pdf]

Supplementary Information (SI) for Physical Chemistry Chemical Physics.
This journal is © the Owner Societies 2026

Supplementary Material: High-resolution spectroscopy of $[H,C,N]^+$: I. Rotationally resolved vibrational bands of HCN^+ and HNC^+

Philipp C. Schmid, Samuel J. P. Marlton, Weslley G. D. P. Silva, Thomas Salomon, János Sarka, Sven Thorwirth, Oskar Asvany, and Stephan Schlemmer*

I. Physikalisches Institut der Universität zu Köln, Köln, Germany

E-mail: schlemmer@ph1.uni-koeln.de

Renner-Teller splitting for HCN^+

In section 4.3 of the main article, the Renner-Teller splitting of HCN^+ is discussed. In particular, Tarroni et al.[1] calculated the fundamental excitation of the ν_2 bending vibration to be at 294.1 cm^{-1} and 821.5 cm^{-1}. Using these values we were able to estimate the bending frequency ω_2 and the Renner-Teller parameter ϵ.

For one quanta in the ν_2 bending vibration, ω_2 and ϵ were calculated, ignoring the spin-orbit coupling in HCN^+, using the vibronic energy G(ν_2,K), as given in Herzberg at al.,[2] with $\nu_2 = 1$ and K = 0 (Σ state):

$$G^{\pm}(\nu_2 = 1, K = 0) = \omega_2\sqrt{1 \pm \epsilon} \cdot (\nu_2 + 1) \tag{1}$$

Thereby $G^+(\nu_2, K)$ denotes the upper Renner-Teller component while $G^-(\nu_2, K)$ the lower Renner-Teller component.

Here the zero point energy contribution in the vibronic energy was taken into account using the following expression[2] and taking $\nu_2 = 0$ and K = 1 (Π state):

$$G(\nu_2 = 0, K = 1) = \omega_2[(\nu_2 + 1) - \frac{1}{8}\epsilon^2 K(K + 1)] \tag{2}$$

Line list for HCN^+

ν_1 fundamental stretching band

Truncated fit file from PGOPHER. Transitions are given in units of cm^{-1}. L denotes the Λ component, while F indicates the Ω state.

Observed	Calculated	Obs-Calc	J'	L'	F'	J"	L"	F"
3030.8664	3030.8668	-0.0004	8.5	f	1	9.5	f	1
3030.8787	3030.8791	-0.0005	8.5	e	1	9.5	e	1
3033.6739	3033.6739	0.0000	7.5	f	1	8.5	f	1
3033.6859	3033.6857	0.0002	7.5	e	1	8.5	e	1
3036.4608	3036.4604	0.0003	6.5	f	1	7.5	f	1
3036.4714	3036.4711	0.0003	6.5	e	1	7.5	e	1

3037.6093	3037.6087	0.0006	5.5	e	2	6.5	e	2
3037.6190	3037.6186	0.0005	5.5	f	2	6.5	f	2
3039.2266	3039.2263	0.0003	5.5	f	1	6.5	f	1
3039.2359	3039.2354	0.0004	5.5	e	1	6.5	e	1
3040.4811	3040.4809	0.0002	4.5	e	2	5.5	e	2
3040.4854	3040.4854	-0.0000	4.5	f	2	5.5	f	2
3041.9716	3041.9714	0.0002	4.5	f	1	5.5	f	1
3041.9790	3041.9788	0.0002	4.5	e	1	5.5	e	1
3043.3372	3043.3375	-0.0003	3.5	e	2	4.5	e	2
3043.3372	3043.3367	0.0006	3.5	f	2	4.5	f	2
3044.6956	3044.6958	-0.0002	3.5	f	1	4.5	f	1
3044.7010	3044.7013	-0.0003	3.5	e	1	4.5	e	1
3046.1715	3046.1718	-0.0004	2.5	f	2	3.5	f	2
3046.1778	3046.1781	-0.0003	2.5	e	2	3.5	e	2
3047.3999	3047.3996	0.0003	2.5	f	1	3.5	f	1
3047.4035	3047.4032	0.0003	2.5	e	1	3.5	e	1
3048.9904	3048.9906	-0.0002	1.5	f	2	2.5	f	2
3049.0013	3049.0020	-0.0007	1.5	e	2	2.5	e	2
3050.0833	3050.0830	0.0002	1.5	f	1	2.5	f	1
3050.0845	3050.0850	-0.0005	1.5	e	1	2.5	e	1
3051.7914	3051.7924	-0.0009	0.5	f	2	1.5	f	2
3051.8077	3051.8085	-0.0008	0.5	e	2	1.5	e	2
3055.7438	3055.7441	-0.0004	3.5	e	2	3.5	f	2
3055.8276	3055.8275	0.0000	2.5	e	2	2.5	f	2
3055.8706	3055.8707	-0.0001	8.5	e	1	8.5	f	1
3055.8940	3055.8944	-0.0004	1.5	e	2	1.5	f	2
3055.9064	3055.9063	0.0001	3.5	f	2	3.5	e	2
3055.9445	3055.9440	0.0005	0.5	e	2	0.5	f	2
3055.9542	3055.9544	-0.0002	2.5	f	2	2.5	e	2
3055.9871	3055.9883	-0.0012	0.5	f	2	0.5	e	2
3056.0369	3056.0374	-0.0005	8.5	f	1	8.5	e	1
3056.0513	3056.0518	-0.0005	7.5	e	1	7.5	f	1
3056.1709	3056.1710	-0.0001	7.5	f	1	7.5	e	1
3056.2099	3056.2100	-0.0001	6.5	e	1	6.5	f	1
3056.2908	3056.2910	-0.0002	6.5	f	1	6.5	e	1
3056.3456	3056.3456	-0.0000	5.5	e	1	5.5	f	1
3056.3973	3056.3971	0.0002	5.5	f	1	5.5	e	1
3056.4589	3056.4587	0.0002	4.5	e	1	4.5	f	1
3056.4888	3056.4886	0.0002	4.5	f	1	4.5	e	1
3056.5499	3056.5500	-0.0000	3.5	e	1	3.5	f	1
3056.5651	3056.5651	0.0000	3.5	f	1	3.5	e	1

3056.6196	3056.6198	-0.0002	2.5	e	1	2.5	f	1
3056.6258	3056.6259	-0.0000	2.5	f	1	2.5	e	1
3056.6685	3056.6687	-0.0002	1.5	e	1	1.5	f	1
3056.6700	3056.6703	-0.0002	1.5	f	1	1.5	e	1
3060.0909	3060.0903	0.0006	1.5	e	2	0.5	e	2
3060.1167	3060.1169	-0.0002	1.5	f	2	0.5	f	2
3062.8186	3062.8184	0.0001	2.5	e	2	1.5	e	2
3062.8465	3062.8468	-0.0003	2.5	f	2	1.5	f	2
3063.2077	3063.2070	0.0007	2.5	e	1	1.5	e	1
3063.2099	3063.2096	0.0003	2.5	f	1	1.5	f	1
3065.5279	3065.5267	0.0012	3.5	e	2	2.5	e	2
3065.5564	3065.5557	0.0006	3.5	f	2	2.5	f	2
3065.7764	3065.7762	0.0001	3.5	e	1	2.5	e	1
3065.7817	3065.7816	0.0001	3.5	f	1	2.5	f	1
3068.2154	3068.2145	0.0010	4.5	e	2	3.5	e	2
3068.2435	3068.2432	0.0003	4.5	f	2	3.5	f	2
3068.3278	3068.3280	-0.0002	4.5	e	1	3.5	e	1
3068.3372	3068.3373	-0.0001	4.5	f	1	3.5	f	1
3070.8631	3070.8628	0.0003	5.5	e	1	4.5	e	1
3070.8774	3070.8770	0.0003	5.5	f	1	4.5	f	1
3070.8819	3070.8814	0.0005	5.5	e	2	4.5	e	2
3070.9088	3070.9086	0.0002	5.5	f	2	4.5	f	2
3073.3807	3073.3808	-0.0001	6.5	e	1	5.5	e	1
3073.4009	3073.4012	-0.0002	6.5	f	1	5.5	f	1
3073.5274	3073.5269	0.0005	6.5	e	2	5.5	e	2
3073.5524	3073.5516	0.0008	6.5	f	2	5.5	f	2
3075.8820	3075.8824	-0.0005	7.5	e	1	6.5	e	1
3075.9095	3075.9100	-0.0005	7.5	f	1	6.5	f	1
3076.1511	3076.1509	0.0001	7.5	e	2	6.5	e	2
3076.1717	3076.1719	-0.0002	7.5	f	2	6.5	f	2
3078.3673	3078.3678	-0.0005	8.5	e	1	7.5	e	1
3078.4029	3078.4036	-0.0006	8.5	f	1	7.5	f	1
3078.7528	3078.7531	-0.0002	8.5	e	2	7.5	e	2
3078.7694	3078.7693	0.0001	8.5	f	2	7.5	f	2
3080.8369	3080.8370	-0.0001	9.5	e	1	8.5	e	1
3080.8820	3080.8822	-0.0002	9.5	f	1	8.5	f	1
3081.3321	3081.3332	-0.0011	9.5	e	2	8.5	e	2
3081.3430	3081.3437	-0.0006	9.5	f	2	8.5	f	2
3083.2907	3083.2902	0.0005	10.5	e	1	9.5	e	1
3083.3458	3083.3457	0.0001	10.5	f	1	9.5	f	1
3085.7277	3085.7274	0.0003	11.5	e	1	10.5	e	1

3085.7953	3085.7942	0.0011	11.5	f	1	10.5	f	1

$\nu_1 + \nu_2$ stretching band

Truncated fit file from PGOPHER. Transitions are given in units of cm^{-1}. L denotes the Λ component, while F indicates the Ω state.

Observed	Calculated	Obs-Calc	J'	L'	F'	J"	L"	F"
3318.8392	3318.8393	-0.0001	3.5	e	1	2.5	e	2
3318.8552	3318.8553	-0.0001	2.5	f	2	2.5	e	2
3320.0880	3320.0879	0.0001	4.5	e	1	3.5	e	2
3320.1084	3320.1085	-0.0001	3.5	f	2	3.5	e	2
3321.3023	3321.3023	0.0000	5.5	e	1	4.5	e	2
3321.3273	3321.3274	-0.0001	4.5	f	2	4.5	e	2
3321.7010	3321.7016	-0.0006	1.5	f	2	0.5	f	2
3322.4848	3322.4845	0.0003	6.5	e	1	5.5	e	2
3322.5142	3322.5142	-0.0001	5.5	f	2	5.5	e	2
3323.6369	3323.6371	-0.0002	7.5	e	1	6.5	e	2
3323.6715	3323.6714	0.0001	6.5	f	2	6.5	e	2
3324.7640	3324.7629	0.0011	8.5	e	1	7.5	e	2
3324.8018	3324.8018	0.0000	7.5	f	2	7.5	e	2
3325.7481	3325.7477	0.0003	2.5	f	2	1.5	f	2
3325.8640	3325.8649	-0.0009	9.5	e	1	8.5	e	2
3325.9086	3325.9084	0.0002	8.5	f	2	8.5	e	2
3343.3510	3343.3507	0.0003	5.5	e	1	6.5	e	1
3346.7886	3346.7887	-0.0001	4.5	e	1	5.5	e	1
3350.3259	3350.3259	0.0000	3.5	e	1	4.5	e	1
3353.9644	3353.9645	-0.0001	2.5	e	1	3.5	e	1
3357.7066	3357.7064	0.0001	1.5	e	1	2.5	e	1
3359.7478	3359.7481	-0.0003	6.5	e	1	6.5	f	1
3359.7785	3359.7778	0.0007	5.5	f	2	6.5	f	1
3360.4607	3360.4608	-0.0001	5.5	e	1	5.5	f	1
3360.4855	3360.4860	-0.0005	4.5	f	2	5.5	f	1
3361.2689	3361.2687	0.0003	4.5	e	1	4.5	f	1
3361.2887	3361.2893	-0.0006	3.5	f	2	4.5	f	1
3361.5537	3361.5533	0.0005	0.5	e	1	1.5	e	1
3362.1745	3362.1746	-0.0000	3.5	e	1	3.5	f	1
3362.1901	3362.1906	-0.0005	2.5	f	2	3.5	f	1
3363.1820	3363.1810	0.0009	2.5	e	1	2.5	f	1
3363.1927	3363.1924	0.0002	1.5	f	2	2.5	f	1
3364.2904	3364.2902	0.0003	1.5	e	1	1.5	f	1

3364.2969	3364.2970	-0.0001	0.5	f	2	1.5	f	1
3369.7684	3369.7682	0.0002	2.5	e	1	1.5	e	1
3369.7792	3369.7797	-0.0004	1.5	f	2	1.5	e	1
3371.4010	3371.4008	0.0001	3.5	e	1	2.5	e	1
3371.4166	3371.4168	-0.0002	2.5	f	2	2.5	e	1
3373.1381	3373.1380	0.0001	4.5	e	1	3.5	e	1
3373.1583	3373.1586	-0.0002	3.5	f	2	3.5	e	1
3374.9778	3374.9780	-0.0002	5.5	e	1	4.5	e	1
3375.0032	3375.0032	0.0000	4.5	f	2	4.5	e	1
3376.9487	3376.9486	0.0001	5.5	f	2	5.5	e	1
3378.0003	3378.0005	-0.0003	2.5	f	2	1.5	f	1
3378.9574	3378.9581	-0.0007	7.5	e	1	6.5	e	1
3378.9932	3378.9924	0.0007	6.5	f	2	6.5	e	1
3382.3749	3382.3751	-0.0002	3.5	f	2	2.5	f	1
3386.8518	3386.8519	-0.0001	4.5	f	2	3.5	f	1

Line list for HNC^+

ν_1 fundamental stretching band

Truncated fit file from PGOPHER. Transitions are given in units of cm^{-1}. S denotes the spin-doublet, while F indicates the Ω state.

Observed	Calculated	Obs-Calc	J'	S'	F'	J"	S"	F"
3372.0542	3372.0527	0.0015	10.5	e	1	11.5	e	1
3372.0542	3372.0573	-0.0031	9.5	f	1	10.5	f	1
Blend	3372.0549	-0.0007						
3375.4298	3375.4278	0.0020	9.5	e	1	10.5	e	1
3375.4298	3375.4322	-0.0024	8.5	f	1	9.5	f	1
Blend	3375.4299	-0.0001						
3378.7818	3378.7801	0.0016	8.5	e	1	9.5	e	1
3378.7846	3378.7844	0.0002	7.5	f	1	8.5	f	1
3382.1115	3382.1097	0.0019	7.5	e	1	8.5	e	1
3382.1115	3382.1138	-0.0022	6.5	f	1	7.5	f	1
Blend	3382.1116	-0.0000						
3385.4185	3385.4162	0.0023	6.5	e	1	7.5	e	1
3385.4185	3385.4202	-0.0017	5.5	f	1	6.5	f	1
Blend	3385.4181	0.0004						
3388.7012	3388.6998	0.0014	5.5	e	1	6.5	e	1
3388.7012	3388.7036	-0.0024	4.5	f	1	5.5	f	1

Blend	3388.7015	-0.0004						
3388.7174	3388.7186	-0.0012	5.5	e	1	5.5	f	1
3391.9620	3391.9602	0.0018	4.5	e	1	5.5	e	1
3391.9620	3391.9638	-0.0018	3.5	f	1	4.5	f	1
Blend	3391.9618	0.0002						
3391.9763	3391.9761	0.0002	4.5	e	1	4.5	f	1
3395.1981	3395.1974	0.0007	3.5	e	1	4.5	e	1
3395.1981	3395.2009	-0.0028	2.5	f	1	3.5	f	1
Blend	3395.1989	-0.0008						
3395.2097	3395.2104	-0.0007	3.5	e	1	3.5	f	1
3398.4124	3398.4146	-0.0021	1.5	f	1	2.5	f	1
3398.4124	3398.4113	0.0012	2.5	e	1	3.5	e	1
Blend	3398.4126	-0.0002						
3398.4207	3398.4214	-0.0007	2.5	e	1	2.5	f	1
3401.6023	3401.6017	0.0005	1.5	e	1	2.5	e	1
3401.6061	3401.6049	0.0013	0.5	f	1	1.5	f	1
3401.6092	3401.6090	0.0002	1.5	e	1	1.5	f	1
3404.7695	3404.7687	0.0008	0.5	e	1	1.5	e	1
3404.7742	3404.7731	0.0011	0.5	e	1	0.5	f	1
3411.0322	3411.0347	-0.0024	1.5	e	1	0.5	e	1
3411.0322	3411.0306	0.0017	0.5	f	1	0.5	e	1
Blend	3411.0333	-0.0011						
3414.1284	3414.1306	-0.0022	2.5	e	1	1.5	e	1
3417.1929	3417.1932	-0.0003	2.5	f	1	2.5	e	1
3417.2010	3417.2028	-0.0018	3.5	e	1	2.5	e	1
3420.2405	3420.2388	0.0017	3.5	f	1	3.5	e	1
3420.2514	3420.2490	0.0024	3.5	f	1	2.5	f	1
3420.2528	3420.2511	0.0017	4.5	e	1	3.5	e	1
3423.2602	3423.2606	-0.0003	4.5	f	1	4.5	e	1
3423.2746	3423.2756	-0.0010	5.5	e	1	4.5	e	1
3423.2746	3423.2736	0.0010	4.5	f	1	3.5	f	1
Blend	3423.2747	-0.0001						
3426.2577	3426.2583	-0.0007	5.5	f	1	5.5	e	1
3426.2756	3426.2743	0.0014	5.5	f	1	4.5	f	1
3426.2756	3426.2761	-0.0005	6.5	e	1	5.5	e	1
Blend	3426.2753	0.0004						
3429.2306	3429.2321	-0.0015	6.5	f	1	6.5	e	1
3429.2522	3429.2510	0.0012	6.5	f	1	5.5	f	1
3429.2522	3429.2526	-0.0005	7.5	e	1	6.5	e	1
Blend	3429.2518	0.0003						
3432.1815	3432.1818	-0.0003	7.5	f	1	7.5	e	1

3432.2040	3432.2036	0.0004	7.5	f	1	6.5	f	1
3432.2040	3432.2051	-0.0011	8.5	e	1	7.5	e	1
Blend	3432.2044	-0.0004						
3435.1330	3435.1321	0.0009	8.5	f	1	7.5	f	1
3435.1330	3435.1334	-0.0004	9.5	e	1	8.5	e	1
Blend	3435.1328	0.0002						
3438.0376	3438.0376	-0.0001	10.5	e	1	9.5	e	1
3438.0376	3438.0365	0.0011	9.5	f	1	8.5	f	1
Blend	3438.0371	0.0005						

References

(1) Tarroni, R.; Mitrushenkov, A.; Palmieri, P.; Carter, S. Energy levels of HCN^+ and DCN^+ in the vibronically coupled $X^2\Pi$ and $A^2\Sigma^+$ states. *The Journal of Chemical Physics* **2001**, *115*, 11200–11212.

(2) Herzberg, G. *Molecular Spectra and Molecular Structure. Vol.III: Electronic Spectra and Electronic Structure of Polyatomic Molecules*; 1950; Vol. 3.
